# The dataset for antifeedant activity of eugenol derived compounds against red palm weevil (*Rhynchophorus ferrugineus*, Olivier) larvae

**DOI:** 10.1016/j.dib.2019.104227

**Published:** 2019-07-08

**Authors:** Tay Karh Yan, Asnuzilawati Asari, Samsuri Abdullah, Marzuki Ismail, Wahizatul Afzan Azmi

**Affiliations:** a School of Marine & Environmental Sciences, Universiti Malaysia Terengganu, 21030, Kuala Nerus, Terengganu, Malaysia; b School of Fundamental Science, Universiti Malaysia Terengganu, 21030, Kuala Nerus, Terengganu, Malaysia

**Keywords:** Red palm weevil, Antifeedant activity, Eugenol, Biopesticide, Coconut pest

## Abstract

*Rhynchophorus ferrugineus* or red palm weevil (RPW) is a destructive insect pest of major cultivated palms such as coconut, date and oil palm. One of the control management of RPW is trunk injection using monocrotophos or methamidophos, but these chemicals are found to affect ecosystems and human health. Thus, we aimed to determine a bio-pesticide to replace these synthetic chemicals. We tested the antifeedant activity of three eugenol-based compounds as potential control agent against RPW larvae in vitro condition for two weeks. All these compounds show significant effect as feeding deterrent agent on 4th instar larvae, while WN16 (4-allyl-2-methoxy-1-(4-trifluoromethyl-benzyloxy)-benzene) shows the highest feeding deterrent index (FDI = 64.42%). Here we present the data regarding the biological aspect on treated RPW larvae as well as antifeedant activity index of these eugenol derived compounds.

**Table:** Specifications table

Subject area	*Agricultural and Biological Sciences*
More specific subject area	*Insect Science*
Type of data	*Table, figure*
How data was acquired	*No choice feeding contact bioassay, weighted using Electronic balance 0.*01 gm *RADWAG WTB 2000*
Data format	*Analyzed*
Experimental factors	*Three replicates of 4*^*th*^*instar RPW larvae were treated with sago palm stems as food diet. Food diet were cut and soaked in each eugenol derived compound with concentrations of* 200 ppm*,* 400 ppm *and* 600 ppm*, while food diet for control was treated with acetone alone.*
Experimental features	*Toxicity of three eugenol derived compounds were tested against RPW larvae for 14 days. Interpretations of data included amount of daily consumption, relative growth rate (RGR), relative consumption rate (RCR), feeding deterrent index (FDI) and multiple range tests (MRT).*
Data source location	*Ecology Laboratory, School of Marine & Environmental Sciences, Universiti Malaysia Terengganu, Terengganu, Malaysia.*
Data accessibility	*Data available within this article.*

**Table:** 

**Value of the data** • These data show the potential effects of eugenol as a feeding deterrent agent against RPW larvae, also help in understanding the function of eugenol in pest control management. • Interpolation of concentration for these compounds show the lowest amount in compounds required to reduce RPW larvae feeding rate, possible to act as a stepping stone to determine effect of similar compounds in the future. • These data could be used as reference or comparison to other studies related to antifeedant activity as well as biological aspect of RPW larvae.

## 1 Data

**Fig. 1:**
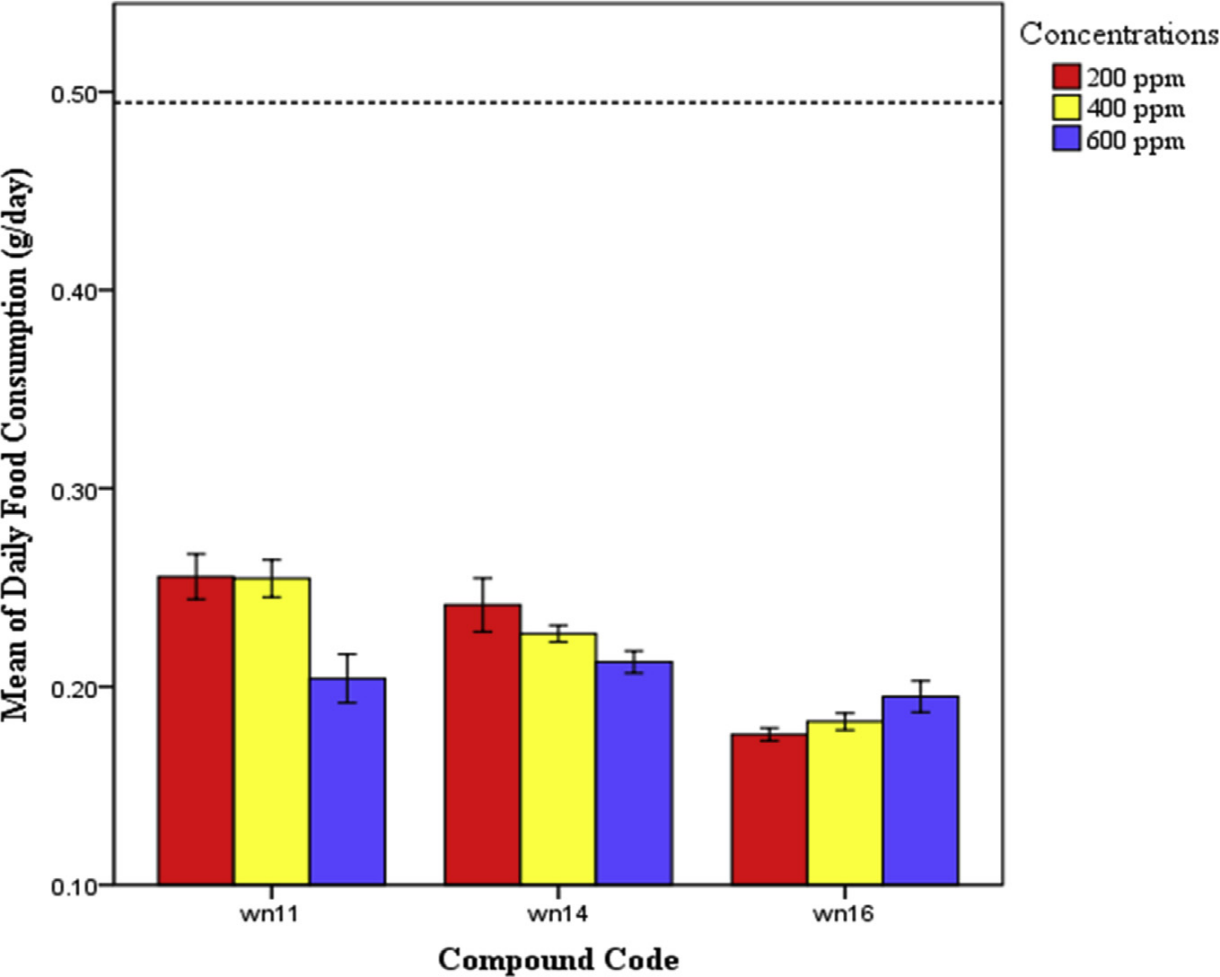
Mean ± SE of daily food consumption (g) of RPW larvae against each derived compound with different concentrations (ppm). Dotted line in the graph indicates value of daily food consumption (g) in control.

In this report, we present the data of daily food consumption (g) versus concentrations of three derived compounds (Fig. 1), mean ± SE values of daily consumptions, relative growth rate (RGR), relative consumption rate (RCR), food ingestion efficiency (ECI), feeding deterrent index (FDI) (Table 1) and result for multiple range tests (Table 2).

**Table 1:** Mean ± SE data values of daily consumptions, relative growth rate (RGR), relative consumption rate (RCR) and feeding deterrent index (FDI). Control were treated with acetone alone. There are three replications that were conducted for each independent experiment.

Treatment	Concentrations (ppm)	Daily consumption (g/day)	RGR (g g^−1^day^−1^)	RCR (g g^−1^day^−1^)	FDI (%)
Control	0	0.4945 ± 0.004^d^	0.0054 ± 0.016	0.4701 ± 0.075^b^	–
WN11	200	0.2555 ± 0.011^b,A^	−0.0158 ± 0.021	0.1117 ± 0.023^a^	48.34^b,A^
	400	0.2545 ± 0.009^c,A^	−0.0060 ± 0.016	0.1163 ± 0.014^a^	48.53^c,A^
	600	0.2040 ± 0.012^a,B^	−0.0018 ± 0.028	0.1051 ± 0.026^a^	58.74^a,B^
WN14	200	0.2412 ± 0.013^b,A^	0.0154 ± 0.013	0.1298 ± 0.016^a^	51.22^b,A^
	400	0.2267 ± 0.004^b,A^	0.0252 ± 0.029	0.1642 ± 0.042^a^	54.16^b,A^
	600	0.2124 ± 0.005^a,A^	0.0123 ± 0.006	0.1469 ± 0.056^a^	57.05^a,A^
WN16	200	0.1760 ± 0.003^a,A^	−0.0019 ± 0.007	0.0642 ± 0.004^a^	64.42^a,A^
	400	0.1824 ± 0.004^a,A^	−0.0097 ± 0.004	0.0529 ± 0.002^a^	63.12^a,A^
	600	0.1950 ± 0.008^a,A^	−0.0119 ± 0.002	0.0791 ± 0.002^a^	60.57^a,A^

*The same small letter indicates no significant difference between derived compounds (p > 0.05).

*The same capital letter indicates no significant difference between concentrations in a same derived compound (p > 0.05).

*No capital letter in whole column shows no significant difference between control and all treatments.

**Table 2:** Multiple Range Tests with 95.0% LSD.

Compound Code	Count	Mean	Homogeneous Groups
WN11	3	51.87	X
WN14	3	54.1433	X
WN16	3	62.7033	X
Contrast	Sig.	Difference	+/− Limits
WN11 - WN14		−2.27333	7.95431
WN11 - WN16	a	−10.8333	7.95431
WN14 - WN16	a	−8.56	7.95431

a Denotes a statistically significant difference.

All eugenol derived compounds reduce the feeding rate of RPW larvae in all three concentrations i.e. 200 ppm, 400 ppm and 600 ppm respectively, with food consumption reduced by more than 50% comparative with larva consuming normal food (control) of 0.4945 g/day. Among three eugenol derived compounds, WN16 was found to be the most in decreasing the feeding amounts of RPW larvae.

The method currently being used to discriminate among the means is Fisher's least significant difference (LSD) procedure. This table applies a multiple comparison procedure to determine which means are significantly different from others. The bottom half of the output shows the estimated difference between each pair of means. An asterisk has been placed next to 2 pairs, indicating that these pairs show statistically significant differences at the 95.0% confidence level. At the top of the page, two homogenous groups are identified using columns of X's. Within each column, the levels containing X's form a group of means within which there are no statistically significant differences. From Table 2, the effectiveness of antifeedant activity of WN16 is significantly different from other compounds.

## 2 Experimental design, materials, and methods

### 2.1 RPW collection & rearing of RPW larvae

Pheromone traps were designed using 7 L polypropylene buckets with four holes perpendicularly cut below the upper rim of the bucket, while the cover had a small knob fixed with screw hook to hang the pheromone sachet (P028 Ferrolure+, 700 mg Lure). 450 g of Morris pineapple slices and 300 ml of tap water were put into each trap as food bait. Pheromone sachet was replaced every three months while food baits were replaced biweekly. These traps were used to collect wild RPWs around coconut plantation areas in Kuala Nerus, Terengganu from September 2017 to December 2018 [1]. Trapped RPWs were collected once a week, then transferred to the laboratory (25 ± 2 °C, 70 ± 5% relative humidity, LD 12: 12 photoperiod) for rearing process. Sugarcane slices were provided as food diet and egg laying substrate for adult RPWs. Neonate larvae or eggs were collected after copulation of adult RPWs. Each larva was transferred into a container and fed with sago palm stem. Instar stages for larva was determined through measurement of head capsule using digital Vernier caliper 0–150 × 0.01 A2583 according to Dyar's ratio and 4th instar larvae were selected for the bioassay experiment. Experiment periods were set in two weeks and three replications were conducted for each treatment with different concentrations.

### 2.2 Preparation of compounds

Eugenol was extracted from clove oil and developed by Dr. Asnuzilawati from School of Fundamental Science, Universiti Malaysia Terengganu. All reactions were performed under nitrogen atmosphere and monitored by thin layer chromatography (TLC) and were visualized under UV 254 nm without treatment. Column chromatography was performed by silica gel 60. Infrared spectra were recorded in KBr disc on PerkinElmer 100 FT-IR spectrometer. UV–visible spectra were recorded on Shimadzu UV-1601 PC spectrophotometer. ^1^H and ^13^C NMR were recorded by Bruker Spectrospin-400 Spectrometer. Elemental analyses were performed on CHNS Analyzer FlashEA 1112 series [2]. Serial dilutions for three eugenol derived compounds were prepared using acetone as solvent to obtain solution in concentrations of 200 ppm, 400 ppm and 600 ppm (Table 3).

**Table 3:** Reference for eugenol derived compounds.

Compound code	Name	Molecular structure	Appearance
WN11	4-allyl-2-methoxy-1-(4-nitrobenzyloxy)-benzene		Yellow solid
WN14	4-allyl-2methoxyphenyl 4-ethylbenzoate		White solid
WN16	4-allyl-2-methoxy-1-(4-trifluoromethyl-benzyloxy)-benzene		White solid

### 2.3 Contact bioassay

Each sago palm stem was cut into block shape in size of 3 cm × 2 cm x 1.5 cm, it was then soaked in 2ml of solution within the Petri dish for 1 min, excessive solution that was not soaked by the food block was removed (approximately 1.4 ml of solution was soaked in each food block). Solvent evaporation process in room temperature was took about 1 h (control was treated with acetone alone). An initial hole was bored for the larva to grub and feed inside the food diet. Each sago palm stem was weighted after evaporation and hole boring process. Each pre-weighted larva was starved for 3 h before the bioassay experiment. Food diet and larva were then transferred to a ventilated plastic container (6 cm diameter x 3.9 cm height). Food diet was replaced daily for two weeks, and at the same time, weight of remained food and weight of larvae were measured and recorded using electronic balance 0.01 gm RADWAG WTB 2000.

### 2.4 Statistical analysis

Calculation of relative growth rate (RGR), relative consumption rate (RCR) and feeding deterrent index (FDI) were based on these formulae:

a. Relativegrowthrate(RGR)=(A−B)/(Bxday)
  A=weightoflarvaafterexperiment;
  B=weightoflarvabeforeexperiment
b. Relativeconsumptionrate(RCR)=D/(Bxday)
  D=weightofthefoodconsumedbylarva
c. Feedingdeterrentindex(FDI)=[(C−T)/C]x100%
  C=Consumedfoodincontrol(weight);
  T=Consumedfoodintreatment(weight)

Significance for daily food consumptions, RGR, RCR and FDI were examined using ANOVA, followed by post hoc Tukey's HSD test in SPSS Statistics version 20. The effectiveness of each derived and significance were determined by multiple range tests with Fisher's least significant difference (LSD) procedure.
